# The role of federal and state policy in addressing early childhood achievement gaps: parent perceptions and student outcomes related to 21st Century Learning Centers programming in the United States

**DOI:** 10.1186/s40723-021-00093-7

**Published:** 2021-11-17

**Authors:** Heather P. Williams

**Affiliations:** College of Education, Boise State University, 1910 University Drive, Boise, ID 83725-1745 USA

**Keywords:** Out of school learning, Parent engagement, Education policy and early childhood education

## Abstract

As policymakers and school communities work to address underlying causes of achievement gaps and access to quality early childhood education, this study considers the use of 21st Century Community Learning Centers to address early childhood education needs on western U.S. state, Idaho. The study sought to understand the relationship between federal and state policies related to out-of-school opportunities to enhance early childhood education. Utilizing data from a statewide evaluation of Idaho’s 21st Century Learning Centers, the study examined 92 centers providing after school, before school, or summer programs in grades preschool through the third grade to predominately at-risk children. Data collection included quantitative data from a survey given to parents (*n* = 183), as well as qualitative data collected through site-based interviews, focus groups and observations. Data included a review of historical and current data on participation rates; attendance rates; standardized test scores for program participants in grades PK-3 (*n* = 3258). Data were analyzed for themes and transfer. The study findings provide further insight into understanding possible relationships between U.S. federal and state policy regarding 21st Century Community Learning Centers on both students’ outcomes and parent satisfaction. The findings further support the role of out-of-school time (OST) experiences in the larger ecosystem of learning and provides insight into understanding how the OST activities are carried over into family life.

## Introduction

As a country, the United States has grappled with how to close the achievement, access and opportunity gaps in our schools, but we also wrestle with our expectation that the traditional school day is the only place where and when children are learning. Decades of research and education policy demonstrate that learning opportunities, family engagement, and development of academic and social–emotional skills also occur after school, before school and during the summer months.

Too many of our children are not ready to gain the skills they need from our educational systems to be successful in their future—either in college or career. They enter school behind, and many will not read at grade level by the end of third grade, or go on to graduate with their peers from high school—not to mention how they might struggle in becoming fully functioning adult citizens able to earn a living to meet a family’s needs. Many of these students are at-risk from an early age and often start school lagging behind in their school readiness, never catching up to become college and career ready. “At-risk” is a term often used to describe students, or groups of students, who are considered to have a higher chance of failing academically because of factors related to their life experiences, race, ethnicity, socio-economic status, and other factors (Elbaum et al., 2000). Underserved learners—often those from racial and ethnic minorities, rural communities, and poverty—are overrepresented in the population of students that are not school ready. Researchers and practitioners alike affirm that in addition to families, peers, and schools, high quality, organized out-of-school time activities have the potential to support and promote youth development. Such activities (a) situate children in safe environments; (b) prevent youth from engaging in delinquent activities; (c) teach youth general and specific skills, beliefs, and behaviors; and (d) provide opportunities for youth to develop relationships with peers and mentors (National Research Council & Institute of Medicine, 2002). However, disadvantaged youth are less likely than their peers to have access to these learning opportunities, and this inequity further deteriorates their chances for school success (Wright, 2011).

The Nita M. Lowey 21st Century Community Learning Centers (21st CCLC) initiative is the only federal funding source dedicated exclusively to supporting local after-school, before-school and summer learning programs. Each 21st CCLC program is shaped by state policies and priorities, as well as the local community of each center, to best meet the needs of the students and organizations it serves. In a meta-analysis by Durlak et al. (2010) evaluating 69 different out-of-school (OST) programs, student participation in OST learning was shown to have an, “overall positive and statistically significant impact on participating youth.” The authors went on to demonstrate that programs that followed the SAFE programming structure (sequenced, active, focused and explicit) saw significant increases in youths’ self perceptions, positive social behaviors, school grades and standardized test scores. Further research (Durlak & Weissberg, 2007; Granger, 2008; Lauer et al., 2006; Vandell, 2013) shows that participation in high-quality OST programs like 21st Century Learning Centers help to close the achievement gap, have positive long-term effects on school attendance and task persistence, have positive effects on school grades and academic work habits, and improves achievement test scores. However, few studies have examined the relationship between 21st CCLC OST programs on early childhood education achievement and family literacy or educational development.

This study examined data from across multiple (*n* = 96) 21st Century Community Learning Centers (CCLC) working to address long-standing achievement and opportunity gaps for their students by providing out-of-school time learning, specifically focusing on those providing early childhood education. All centers in the study were located in Idaho where kindergarten is not mandated, nor is all day kindergarten funded. Pre-school is not considered part of the public school system and is often not available in rural communities. Further, statewide data from the Idaho Reading Indicator shows that less than half of Idaho’s students have the basic skills to enter school and be ready to learn when they come to kindergarten (Richert, 2018). They are not able to recognize basic letters, numbers or colors and many never catch up and read at grade-level by the end of 3rd grade. Lesnick et al. (2010) explain the importance of reading on grade level by third grade, “Third-grade reading level was shown to be a predictor of eighth-grade reading level and ninth-grade course performance even after accounting for demographic characteristics…it is also shown to be a predictor of graduation and college attendance.” The issues often become amplified in high-poverty districts, where students have less access to academic opportunities and vocabulary outside of school, as well as limited options for childcare, quality early childhood education programs, or resources to support parents’ efforts as their child’s first teacher.

The purpose of this study is to examine the influence of OST learning, specifically 21st CCLC programs, on student achievement, as well as explore parent perceptions of the value of the program. The research design utilized data from the administration of Idaho’s statewide assessment for K-3 students known as the Idaho Reading Indicator. The State Department of Education is responsible for collecting and reporting results from the IRI. Staff in local schools record IRI “correct scores” and skill level (i.e., achievement level) for each student in kindergarten through the third grade in the fall and in the spring (Stoneberg, 2016).

Research Question 1: What was the effect of 21st CCLC programs on student performance on the Idaho Reading Indicator for 2017–2018?

Research Question 2: What is the effect of 21st CCLC programming on early childhood education as perceived by parents?

## Review of the literature

We situated our study within three areas of existing research regarding out-of-school learning: education policy providing for 21st CCLC programs, early childhood education impacts on student outcomes, and parent engagement with out-of-school learning programs. In support of our presentation of the literature, we used Bronfenbrenner’s ecological systems theory to explore the interaction of various contexts on the developing child and how the interactions between various systems may influence the child’s learning.

### Bronfenbrenner’s ecological systems theory

Bronfenbrenner’s ecological systems theory (1994) suggests that the various environments that a child interacts with are influenced from within and between other environments, and thus the development of the child is dependent upon many factors. Urie Bronfenbrenner’s initial theory (1979) is grounded in the notion that to understand human development we must study it in the context, or ecology, in which it exists. The theory can be viewed as layers of nested interactions or systems that influences the child’s development, and thus the relationships and interactions between the complex layers also affect the child.

**Fig. 1:**
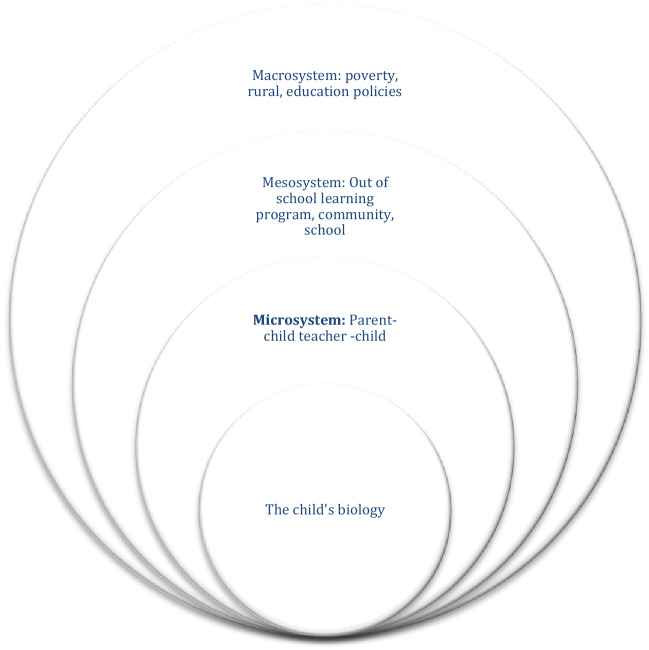
An adaptation of Bronfenbrenner’s ecological systems theory

At the core of the model is the child’s own biology: age, health, temperament, and other biological factors that influence learning—which were not included as a study variable. The next level is the child’s relationship with a parent or parental figure, and teachers or other early childhood care providers, Bronfenbrenner calls this the microsystem. This is the system closest to the child and is a key determinant in a child’s development. The microsystem is nested within the larger mesosystem. Mesosystems might be the family, school, or community, or specific interventions such as early childhood education or out-of-school learning programs. Finally, the largest system in Bronfenbrenner’s theory is the macrosystem which houses social structures, culture, and established policies and laws that may encourage or hinder the child’s development. This study focused on interaction between the microsystem of the parent–child and teacher–child relationships; the mesosystem of the intervention program of early childhood education through out-of-school learning programs; as well as the broader macrosystem of federal policies to address achievement outcomes for at-risk youth (Fig. 1).

### Macrosystem: education policy related to 21st Century Community Learning Centers program

Although the majority of school funding in Idaho comes from state and local resources, the federal role is important in setting the tone for the policy agenda. Federal policy provides the guidance and direction for states and local school districts, especially when it comes to federal funding of programs meant to close the achievement gaps for disadvantaged youth. The 21st Century Community Learning Centers (CCLC) program is currently authorized under Title IV, Part B, of the Elementary and Secondary Education Act (ESEA), as amended by Every Student Succeeds Act (ESSA) of 2015. The program began in the 1990s when the Improving Americas Schools Act was passed to create a federal funding stream for after-school programs. Today’s 21st CCLC program is the result of a dedicated source of federal funding exclusively to support after-school programs that began in 1997, was reauthorized in 2002, and again in 2015. Each state receives funds based on its share of Title I funding for low-income students.

The federal program supports the creation of community learning centers that provide academic enrichment opportunities during non-school hours for children, primarily those who attend high-poverty and low-performing schools. The program is designed to provide academic enrichment opportunities, art, music, recreation, sports, drug and violence prevention, and youth development activities to students during non-school hours. The program also offers families of students served by community learning centers opportunities for educational development and support for family literacy.

### Mesosystems: 21st CCLC and early childhood education in Idaho

A mesosystem is defined by Bronfenbrenner (1979) as a set of interrelations between two or more contexts impacting the child. Since 2003, Idaho has participated in the federal program and received funding from the U.S. Department of Education to operate 21st Century Community Learning Centers (21st CCLCs). The state program is administered by the Idaho State Department of Education (SDE), which provides sub‐grants to support out‐of‐school‐time programs across the state. Individual grantees in turn operate “centers” that provide academic enrichment and other support services or activities for K-12 students and their parents or guardians. Most grantees are local education associations (school districts) and some are community organizations or nonprofit agencies. The U.S. Department of Education has provided funding for 21st CCLC grantees in “rounds;” each round of funding lasts for five years. The 21st CCLC program in Idaho supports community efforts to: (1) help students meet state and local standards in core academic subjects, such as reading and math; (2) offer students a broad array of enrichment activities that can complement their regular academic programs; and (3) offer literacy and other educational services to the families of participating children.

There is much debate in Idaho and across the United States about early childhood education—how to best serve students, particularly those in poverty, and how to prepare them for academic success in and beyond their K-12 schooling. Bassok et al. (2016) point to a number of recent studies showing that children’s academic skills during early childhood—particularly their math skills—are the strongest predictors of their later performance on a number of cognitive and noncognitive outcomes (Claessens & Engel, 2013; Claessens et al., 2009; Duncan et al., 2007; Watts et al., 2014). There is also evidence that exposure to academic content in kindergarten (particularly, engaging and advanced content) can be beneficial for student learning (Claessens et al., in press; Clements & Sarama, 2011; Engel et al., 2015). Magnuson et al. (2007), for example, show that more academically oriented early elementary experiences can help children who did not attend preschool catch up with their peers. Despite research such as this in support of early childhood programming and the need for early intervention, especially for disadvantaged children, Idaho does not require preschool or kindergarten for any student. Kindergarten, which has been identified by the state as an early childhood program, is not required and is not fully funded. Idaho currently only funds a half day of kindergarten. Idaho has accepted the Common Core Standards, now called the Idaho Core, which includes standards to be taught in kindergarten, but yet, Idaho children are not required to attend public school until the age of 7, which is identified as first grade (Idaho Instructional Manual for Reporting Attendance, p. 2). Without state funding for early childhood education many districts have utilized federal funding in the form of Title 1 or 21st CCLC grants to provide support for early learners.

Previous research (Durlak & Weissberg, 2007) has focused on improvements in personal and social skills for students attending 21st CCLC programs. Granger (2008) presents findings about effectiveness of various types of OST programming, but states, “We undoubtedly need good work on the effects of after-school programming.” There is a need to study academic outcomes for early childhood programming, as well as parent perceptions related to the value of the program.

### Microsystem: parent–child interaction with 21st CCLC programs in Idaho

The role of the parent as the first teacher is important to support. Children who lack a stimulating home life during their early development years often show deficits in basic skills upon entering kindergarten (Brown, 2013). Parents in poverty are often not able to provide the same resources and experiences for their children and may lack access to information and materials to support their child’s learning and development. Children who have had rich pre-literacy experiences, such as ongoing reading by parents and encountering a variety of words through everyday conversations have better school outcomes (Dearing et al., 2004). Further research from Jeynes (2012) shows that parent involvement in their child’s school experience and educational development are strong indicators in the success of the child’s learning. Furthermore, parents from low socio-economic backgrounds are less likely to engage in communication with teachers about their children’s early literacy development and how to support children’s learning at home, especially if there are language or cultural differences between the home and school (Sheldon & Epstein, 2005). For these parents, involvement in their children’s education is vital, as research (Dearing et al., 2004) demonstrates that parent involvement has especially strong outcomes for children of low-income families and English Language Learners and children of diverse cultural backgrounds (Jeynes, 2012).

## Research methodology

The study sought to understand potential indicators of effective practice related to the implementation of the 21st Century Community Learning Centers to support early childhood education. The study used data from the statewide evaluation of Idaho’s 21st Century Community Learning Centers, which included all 21st CCLC grantees in Idaho during 2017–2018. Data collection included quantitative data from a survey given to parents, as well as qualitative data collected through site-based interviews, focus groups and observations. Data included a review of historical and current data on participation rates; attendance rates; standardized test scores; and other district measures being targeted by the state grant program and guidelines. We obtained contextual, demographic and historical data through relevant documents and interviews with selected individuals. Finally, the outcomes of individual program evaluations were compared to determine themes, possibilities, and draw broader conclusions on the influence of the 21st CCLC grant on closing achievement and opportunity gaps for school readiness in Idaho.

### Participants

First it is important to discuss the greater macrosystem of Idaho education before looking specifically at the mesosystem of 21st CCLC programs in Idaho. Idaho public schools enroll approximately 300,000 students, in grades PK through 12. The student population is predominately White, with about 12 percent Hispanic, and about 1 percent other categories. Each 21st CCLC program establishes the criteria for student participation. Some programs target specific students based on academics, social–emotional factors, and socio-economic status; however, others may open the program up to any student in the community. The information below characterizes the students served by the 21st CCLC out-of-school program across all sites in Idaho. Data regarding program enrollment and attendance, as well as the demographics of the students served are provided. Students served by the program in Idaho are more diverse than the state average with 63 percent White; 21 percent Hispanic; 5 percent American Indian/ Alaska Native; 4 percent two or more races; and less than 1 percent Asian, Black, Pacific Islander. Note that race and ethnicity data were not available for 5 percent of the students served.

Thirty percent of the students have been identified as being economically disadvantaged; with eleven percent of the students receiving English Language Learners (ELL) services; and eleven percent of the students qualifying for an IEP for Special Education services. More boys than girls are served by after-school programs in Idaho at 46 percent compared to 44 percent (note gender data were not provided for approximately 10 percent of the students served) (Table 1).

**Table 1:** Race and ethnicity of participants

American Indian/Alaska Native	5%
Asian	Less than 1%
Black/African American	Less than 1%
Hispanic/Latino	21%
Native Hawaiian/Pacific Islander	Less than 1%
White	63%
Two or more races	4%
Data not provided	5%

Source: Idaho State Department of Education

Of the total students served by 21st CCLC programs in 2017–2018, over 59 percent are regular program participants (RPP) meaning they attended at least 30 or more days of programming, with over a third of the RPP attending more than 90 days a year (see Table 2). Over 42 percent of students served are early elementary (grades PK-3), less than four (4) percent are high school aged, and the rest (approximately 54 percent) are in grades 4 through 8.

**Table 2:** Other student characteristics for ALL students served by 21st CCLC in Idaho (*n* = 7653)

English language learners	11%
Qualifying for free and/or reduced lunch	30%
Special needs/IEP	11%
Number of family members that participated in programming in 2017–2018	15,713
Males	46%
Females	44%
Gender data not provided	10%

Source: Idaho State Department of Education (2018)

During the 2017–2018 school year there were a total of 96 centers operated under the state’s 21st CCLC program. Of the 96 centers operating in Idaho, four were excluded from the data set because they did not offer early childhood education programming—determined by whether or not they offered programming to students in grades 3 or lower. The focus of this study is on the influence of OST learning on early childhood education, therefore our data concentrated only on the centers (*n* = 92) offering K-3 programming. The student data related to these centers have been previously discussed in this article.

Of the remaining 92 centers we collected data from program staff interviews, program observations, student surveys, and parent surveys from 45 centers that were in their third year of programming. This purposive sample included centers representative of all geographic locations across Idaho. Observation and qualitative notes from interviews were kept in an excel spreadsheet and coded for themes, as well as used for triangulation with the survey data.

The parent perceptions survey was a convenience sample of those that chose to complete the survey (*n* = 183). Parents were given either a link to an online survey or were given the opportunity to complete a paper copy of the survey. All parents had children who were participants in the 21st CCLC centers discussed above. The survey results were downloaded into an excel spreadsheet and analyzed using coding and/or descriptive data.

Finally, student achievement data were analyzed across all 92 centers and included data for 3258 students in grades PK-3. This initial data was cleaned to include only those participants that had both a pre- and post-test score on the Idaho Reading Indicator. If participants were missing either the pre- or post-score their data were deleted from the sample. Once the data were cleaned 1675 students were identified as having both pre- and post-scores. This remaining sample of students (*n* = 1675) were representative of the overall group of students (*n* = 3258) in terms of demographics and student achievement. An excel spreadsheet kept track of student achievement data and analysis looked at those students that were not proficient on the pre-test and how many moved to proficient on the post-test.

Each of the 21st CCLC grants in Idaho focus on three primary areas: attendance, academic improvement, and parent engagement. The requirement for parent engagement sets the grantees apart from other early childhood programs in non-21st CCLC school districts. Across all sites, there were several opportunities for students to continue to learn new skills and discover new opportunities in addition to the regular school day. Parents, staff, and student participants reported the value of the 21st CCLC programs. Through the survey data parents clearly expressed a belief that the programs serve as catalysts for academic improvement as well as for improvement in behaviors, attitudes and efficacy for the students served. All sites reported an absence of local daycares or out-of-school resource centers to help students with academic or social delays. The daycares that did exist in the communities are run as home-based businesses focused on childcare and not necessarily academic enrichment. The districts describe their students as an underserved population at risk due to economic, health, safety, and special needs issues such as ELL, Homelessness, and migrant students. The desire to provide quality early childhood education was the impetus for their 21st CCLC grant applications in each case. All centers identified their desired outcome of providing at least an additional 13 h per week of academic, social–emotional, and recreational enrichment in a constructive, safe environment to help students and their families develop a well-rounded lifestyle of learning and prepare for academic success in meeting the Idaho Core Standards.

The measures in this study include the dependent variables of fall and spring reading scores on the Idaho Reading Indicator, student attendance in the 21st CCLC program, and parental participation in the 21st CCLC program. The independent variables in this study are students are enrolled in grades kindergarten through third grade, race/ethnicity/gender, ESL status, Special education status, and other demographic indicators. The measures related to the Idaho Reading Indicator tests include fall scores that were taken September 2017 and the spring scores represent the assessment taken in May 2018. Students were included in this study if they were enrolled in grades kindergarten through third grade, had a fall and spring IRI score and participated in the 21st CCLC program, regardless of their proficiency level or number of days they attended the program.

## Findings

### Influence of 21st CCLC programs on student performance

The mesosystems explored in this study was that of the family, the early childhood intervention program (21st CCLC), and the school community. The influence of the statewide 21st CCLC programs was assessed based on the percentage of program participants who moved from not meeting proficiency to meeting or exceeding proficiency on the Idaho Reading Indicator (IRI) while regularly attending 21st CCLC programs. The IRI is a statewide assessment for early literacy that focuses on phonemic awareness, alphabetic knowledge, fluency, vocabulary, and comprehension with appropriate subtests at each grade level. Students that had a pre-test and who also had a post-test were examined to see how many made growth from not proficient on the pre-test to meets or exceeds proficiency on the post-test. Overall, across grade levels over 39 percent saw improvement on the Idaho Reading Indicator (see Table 3). Typical growth on the Idaho Reading Indicator is 15 percent from fall to spring for all K-3 Idaho students (Comprehensive Literacy Plan, Idaho SDE, 2020), therefore those students in 21st CCLC programs are outperforming their peers.

**Table 3:** Percentages of 21st CCLC students showing growth pre to post testing

Exam	Total students not proficient on pre-test	Total # of same student population proficient or higher on post-test	Percent of change
IRI (K-3rd grade)	1675	660	39.4%

However, if we breakdown the data to look at it by grade level we see the largest gains for students on the IRI in kindergarten with over 68 percent of all program participants seeing improvements pre to post. Upon further examination, it appears that the more students attend OST programming the larger the gains are in academic achievement. Over 75 percent of kindergarteners that attended over 90 days of programming made gains pre to post. The lowest number of gains was made by third grade students (see Table 4).

**Table 4:** Attendance and academic achievement for students K-3 participating in 21st CCLC programs (total *n* = 1675)

	Attended < 30 days	Attended 30–59 days	Attended 60–89 days	Attended 90 days+
Kindergarten (*n* = 502)				
#not proficient (pre)	64	78	102	258
#improved to proficient or higher (post)	34	40	74	194
First grade (*n* = 320)				
#not proficient (pre)	95	64	71	90
#improved to proficient or higher (post)	30	15	26	29
Second grade (*n* = 457)				
#not proficient (pre)	136	104	98	119
#improved to proficient or higher (post)	35	30	30	44
Third grade (*n* = 396)				
#not proficient (pre)	103	96	89	108
#improved to proficient or higher (post)	23	27	31	31

It is also important to note that the data points above are strictly descriptive data points and not inferential statistics. It is also important to keep in mind that there may be other factors or variables causing or contributing to the gains in achievement for these students that were not studied in this study.

### Parent perceptions of the 21st CCLC programming

A convenience sample of parents from across all geographic regions of Idaho were surveyed regarding their perceptions of the quality of services provided for their children. They were asked to respond to statements with agree; neutral; or disagree. A total of 183 surveys were received from 42 centers offering 21st CCLC programming across Idaho. Overall, parents viewed the program positively with 91 percent agreeing the 21st CCLC program is a benefit to their child. Seventy-six (76) percent agreeing the program addresses their child’s specific needs and 74 percent agreeing the parent activities also met their needs.

## Limitations

This study does further the research on the influence of OST learning, specifically 21st CCLC grants, in both the areas of academic achievement and parent perceptions. We explored the relationship between federal and state policies related to out-of-school opportunities to enhance early childhood education. However, the study is limited by several factors. The first limitation is the use of the Idaho Reading Indicator to measure academic achievement because the IRI is a screening assessment that gives only a “snapshot” of student’s reading ability and was not designed as a comprehensive diagnostic reading assessment (Idaho State Department of Education, 2013). The second limitation is the voluntary nature of the program itself and parent participation in this study, which may or may not have been representative of all parents and students. There is the potential for self-selection bias because of the voluntary nature of program participation. Finally, all centers were included in this data without identification, rating, or controlling for quality of programming offered by centers.

## Conclusions

This study furthers the research in understanding the interplay of both federal and state policy as it relates to OST learning, and specifically the work of 21st Century Community Learning Centers in addressing achievement gaps and access to quality early childhood education. The inclusion of data across multiple centers and all Idaho sites allowed for a deeper understanding in this area and also brought to light opportunities to explore best practices in implementation of early childhood OST programming. Across all centers, over 39 percent of students in grades kindergarten through third grade improved their proficiency level on pre to post tests on the IRI. This does not take into account frequency of attendance, reasons for attendance, types of activities offered, or program quality. Further, this study supports previous research (Holstead & King, 2011; Jenner & Jenner, 2007) in that frequency of OST learning is important to effect academic change. Students that attended ninety days or more saw the greatest level of improvement in proficiency level from pre to post tests in this study. The data support OST learning gives early learners access to programming that supports their academic achievement and ability to read at grade level by the end of first grade. It furthers supports previous research (Lauer et al, 2006; Weiss et al., 2009) showing after-school or OST activities are associated with academic gains for disadvantaged or at-risk youth. Future research could further examine the impact on social–emotional learning and wellness outcomes for early learners, which may further illuminate differences in school readiness and long-term academic attainment.

Bronfenbrenner (1995) found that when a supportive link is formed between various systems this improves the child’s development. The positive potential is further enhanced when the microsystems (parents, teachers, or caregivers) actively participate with each other in these various settings related to the child. In establishing beneficial relationships with the parents, the 21st CCLC program is able to address the needs of the students they are serving, as well as empower parents to meet their children’s needs at home. Based on the parent survey responses (*n* = 183), overwhelmingly parents perceived the 21st CCLC programs as a positive support for students. Centers reported a variety of activities and family/community engagement events, which is an indication of both the level and diverse types of support offered to students and families. Statewide, a total of 27,203 parents and other adults attended 1189 distinct family and community engagement events that were offered by 21st CCLC in 2017–2018 to provide families with meaningful opportunities to be actively engaged in their child’s education. For several decades there has been an increase in both federal and state policy regarding parent involvement and family involvement activities (Weiss et al., 2011), as well as numerous studies on the importance of parenting in early childhood learning (Lareau., 2003; O’Donnell, 2008). The findings of this study support the role of OST experiences in the larger ecosystem of learning and parents desire to have such learning opportunities available to their children. Research (Dearing et al, 2004) also shows how this may be even more important to at-risk children because they benefit more from family involvement in learning than their more affluent or advantaged peers, and these effects tend to persist over time. Future research may further examine which activities are most beneficial to which types of parents, and how the OST family activities are carried over into family life.

Decades of research make clear the need for high-quality early childhood learning opportunities, as well as the opportunities that can be attained from OST learning. This study observes how multiple communities are using federal funding to provide OST learning and early childhood education in a state that does not provide universal preschool or require students to even attend school until age seven. Finally, preliminary reports seem to indicate the widespread learning loss due to the global COVID-19 pandemic is especially concerning among younger children. OST programs, like those in this study, may be beneficial in narrowing the achievement gaps and addressing related issues to learning loss due to the COVID-19 pandemic.

## Data Availability

Available through the Idaho Department of Education.
